# Primary Renal Carcinoid with Bilateral Multiple Clear Cell Papillary Renal Cell Carcinomas

**DOI:** 10.1155/2017/9672368

**Published:** 2017-05-23

**Authors:** Daniel A. Anderson, Maria S. Tretiakova

**Affiliations:** ^1^Department of Pathology, UWMC Anatomic Pathology, University of Washington, 1959 NE Pacific St., P.O. Box 356100, Seattle, WA 98195-6100, USA; ^2^Department of Pathology, University of Washington, 325 Ninth Avenue, P.O. Box 359791, Seattle, WA 98104, USA

## Abstract

Clear cell papillary renal cell carcinoma (CCPRCC) is a newly recognized entity in the 2016 WHO classification and usually presents as a small, circumscribed, solitary mass of indolent nature. CCPRCCs could seldom occur in conjunction with other synchronous or metachronous kidney tumors and even less frequently as bilateral masses. To our knowledge, multiple bilateral CCPRCCs have never been described with the existence of a synchronous well-differentiated neuroendocrine tumor of the kidney and hence reported here as a unique case. This case report highlights the importance in considering this entity and its unusual presentation in the differential diagnosis as a possible mimicker of Von Hippel-Lindau syndrome.

## 1. Case Report

**Figure 1:**
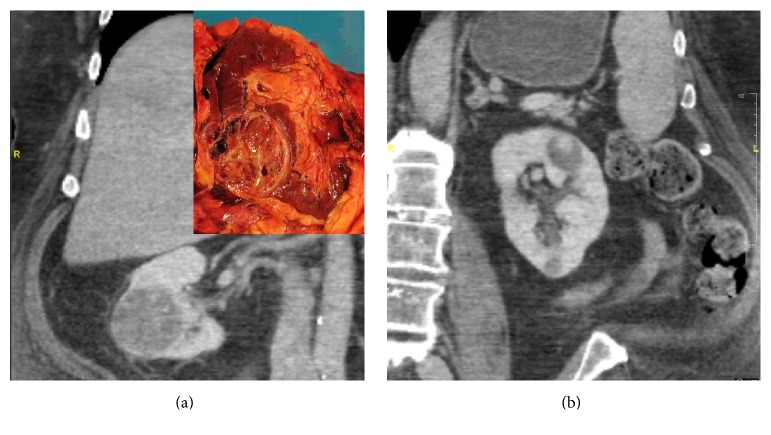
Axial CT images of the (a) right kidney with the largest mid-kidney mass and corresponding gross image [inset] and (b) left kidney with largest upper pole mass.

A 55-year-old male with a notable history of diabetes, hypertension, obesity, and chronic kidney disease was found to have bilateral kidney masses while undergoing ultrasound for evaluation of recurrent kidney infections. He was eventually referred to our institution. Computed Tomography (CT) at that time showed a left upper pole 3.2 cm mass, left lower pole 1.4 cm mass, a right mid-kidney 6.6 cm mass, and an upper pole 2.1 cm mass (Figure 1) which were felt to be stable from a prior CT. A review of a prior biopsy showed small fragments of tissue with CK7 positive, focally CD10 positive clear cells, and mild nuclear atypia which was suspicious for clear cell papillary renal cell carcinoma (CCPRCC).

Resections from the left kidney were performed. The upper pole, lower pole, and the large mid-portion to lower pole masses were removed. All resections showed a histologically typical CCPRCC with a background of simple renal cysts and moderate global glomerular sclerosis, hyperplastic arteriolosclerosis, and lymphoplasmacytic interstitial nephritis.

**Figure 2:**
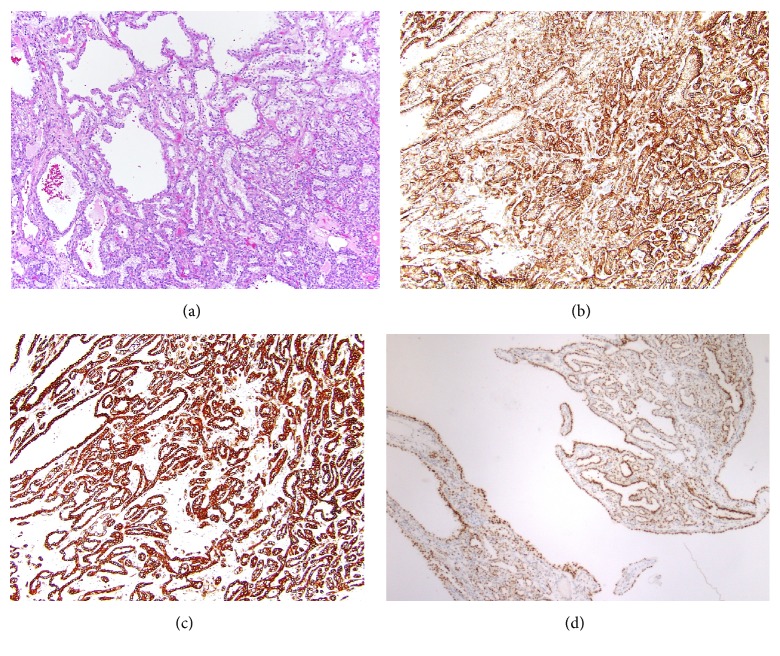
Representative photomicrographs of the CCPRCC component. (a) H&E section shows tubular, papillary, and cystic components of classic CCPRCC with low-grade clear cells in linear alignment (×20) with (b) CAIX showing positivity in a “cup-like” pattern (×20), (c) diffuse positivity for CK7 (×10), and (d) GATA3 showing diffuse staining (×10).

**Figure 3:**
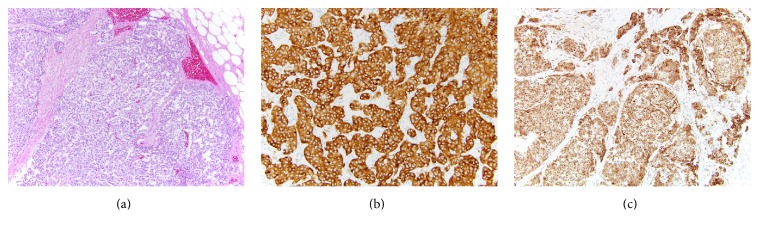
Representative photomicrographs of the well-differentiated neuroendocrine tumor component. (a) H&E section shows the nested pattern of the well-differentiated neuroendocrine cells (×10) with invasion into the hilar fat; (b) synaptophysin (×20) and (c) chromogranin (×10) are diffusely positive.

Several months later, a radical nephrectomy of the right kidney was performed (Figure 1, inset). Pathologic evaluation showed a 6.5 cm CCPRCC, ISUP (International Society of Urological Pathology) grade 2, of the mid-lower pole which was limited to the kidney (Figure 2). These cells were positive for carbonic anhydrase IX (CAIX) in a cup-like staining pattern, cytokeratin 7 (CK7), and paired box 8 (PAX8) and were negative for neuroendocrine markers synaptophysin and chromogranin. Interestingly, GATA3 also showed diffuse strong positivity of CCPRCC. No abnormalities of* Von Hippel-Lindau (VHL)* gene, including mutations, methylation abnormalities, and LOH3p, were identified by genetic testing at an outside institution. Just adjacent to the CCPRCC, a microscopically separate 1.5 cm well-differentiated neuroendocrine tumor was identified focally invading the hilar fat (Figure 3). This was diffusely positive for synaptophysin and chromogranin, variably positive for CK7, and negative for CAIX, PAX8, and GATA3. Of note, no symptoms of carcinoid syndrome were present. In addition to the above histologic findings, simple 0.5–1.5 cm cortical cysts were identified in a background of focal global glomerulosclerosis with tubular atrophy, interstitial fibrosis, and moderate to severe arteriosclerosis.

One year after his partial nephrectomy, the patient is doing well with no evidence of recurrence. The patient's chronic kidney disease is stable with creatinine of 1.81 mg/dL (0.8 mg/dL prior to surgery) and does not require dialysis.

## 2. Discussion

CCPRCC was described less than a decade ago [1] as a distinct entity representing 1–4% of renal epithelial neoplasms [2, 3]. It was recently recognized in the 2016 WHO classification [4]. CCPRCC is composed of low-grade clear cells (ISUP grade 1 or 2), which contain nuclei polarized away from the basement membrane and a wide range of architectural components including papillary, cystic, acinar nests, ribbons, and solid components. Immunohistochemically, CCPRCC is positive for diffuse CK7 membranous staining, CAIX staining in a “cup-like” pattern, and is negative for TFE3, CD10, and alpha methyl acyl coenzyme A racemase (AMACR) [5, 6]. The literature has been mixed on the expression of GATA3 in CCPRCCs. Some sources report it as negative [7] while others report CCPRCCs as positive [8]. One study reported that up to one-third of CCPRCCs are positive for GATA3, though staining is reported to be moderate and to be seen in only 10% of the tumor cells [9]. Analysis of miRNA has shown overexpression of the miR-200 family, a regulator of the epithelial-mesenchymal transition (EMT), which is reflected in CCPRCC's positivity for E-cadherin, vimentin, and *β*-catenin and may play a role in CCPRCCs indolent nature [10]. Interestingly, neuroendocrine neoplasms have shown overexpression of the miR-200 family in well-differentiated small intestinal neuroendocrine tumors [11]. In addition, GATA3 expression is found to correlate with elevated E-cadherin levels and plays a role in the reversal of EMT [12–14]. The occurrence of the well-differentiated neuroendocrine tumor and the coexisting CCPRCC may be related to miR-200 family overexpression.

Though first described in patients with end stage renal disease [15], the majority of CCPRCCs have been found in those with healthy kidneys [16, 17]. CCPRCC occurs in a wide range of patients' age (18 to 88 years) with a mean age of 70. Tumors can range from 0.3 to 7.5 cm with a mean of 2 cm and a majority being pT1a. Multifocal tumors have been described including multiple ipsilateral foci, bilateral foci, and separate synchronous tumors including clear cell renal cell carcinoma, multilocular cystic renal cell carcinoma, oncocytoma, papillary adenomas, papillary renal cell carcinoma, and acquired cystic kidney disease associated renal cell carcinoma [18–21]. Though data are limited, current evidence supports the conclusion that CCPRCC have an excellent prognosis and indolent course. There are no convincing reports of recurrences, metastases, or sarcomatoid transformation after surgical excision. Likewise, multifocality and bilaterality do not appear to impart a worse prognosis [2, 22].

Awareness of this newly described combination of CCPRCC and neuroendocrine tumors is critical to consider in the differential diagnosis of VHL syndrome. VHL syndrome can present with multiple clear cell renal cell carcinomas which can mimic CCPRCC morphologically and grossly. However, these tumors differ in their molecular and immunohistochemical profiles [23]. In addition, VHL disease may present with endocrine manifestations including pheochromocytomas, extra-adrenal paragangliomas, and pancreatic neuroendocrine tumors [24]. Though VHL syndrome was a consideration with the constellation of findings described in this case, this was effectively ruled out through immunohistochemistry and genetic testing. Scant evidence suggests that CCPRCC harbors* VHL* mutations and the classification of such CCPRCC cases with* VHL* mutations is questionable [10, 25–27].

Primary renal carcinoid tumors are extremely rare. Peak incidence is the fifth and sixth decades of life with a slight female predominance. Renal carcinoids have been associated with horseshoe kidneys and polycystic kidney disease and have been described arising from teratomas, including one case report of a reported adenocarcinoma and a carcinoid arising within a teratoma in a horseshoe kidney [28, 29]. The neoplasm commonly shows trabecular, pseudoglandular, solid patterns and immunophenotypically expresses neuroendocrine markers [30]. Most carcinoids have an indolent course despite their frequent presentation with regional metastasis to the lymph nodes and documented distant metastases to liver, lungs, and bones [31, 32]. Carcinoids have been associated with loss of heterozygosity of the 3p12-3p21 region and higher rates of metastases have been reported with tumors greater than 4 cm and with higher mitoses [33]. In our case, a paraganglioma of the kidney was initially entertained. However, morphological assessment as well as lack of GATA3, cytokeratin, and S100 staining of sustentacular cells effectively ruled this out.

CCPRCC has never before been reported to coexist with a primary renal carcinoid tumor much less with bilateral multiple CCPRCCs. Though CCPRCCs have an indolent course, multifocality and synchronous tumors of various types, including well-differentiated neuroendocrine tumors, as described here, must be considered in the prognosis and raise the concern of possible syndromic associations. Further studies are needed to better understand these neoplasms, their interaction, and their underlying mechanisms.

## 3. Conclusion

This is an unprecedented case of a primary renal carcinoid with simultaneous multifocal bilateral CCPRCCs. The presence of concurrent multiple tumors, besides their negative impact on residual kidney function, should be considered in the diagnosis in the otherwise indolent prognosis of CCPRCC. This case report highlights the importance in considering this entity and its unusual presentation in the differential diagnosis as a possible mimicker of Von Hippel-Lindau syndrome.
